# Prevalence and Impact of Sarcopenia in Chronic Pancreatitis: A Review of the Literature

**DOI:** 10.1007/s00268-020-05828-0

**Published:** 2020-11-09

**Authors:** Li Lian Kuan, Ashley R. Dennison, Giuseppe Garcea

**Affiliations:** 1 Department of Hepatobiliary and Pancreatic Surgery, University Hospitals of Leicester NHS Trust, Gwendolen Road, Leicester, LE5 4PW UK; 2 Discipline of Surgery, The Queen Elizabeth Hospital, University of Adelaide, Adelaide, SA Australia

## Abstract

**Introduction:**

Malnutrition is a common sequela of chronic pancreatitis (CP). Alterations in body composition and the assessment of sarcopenia have gained the interest of clinicians in recent years. There is a scarcity of data currently available concerning sarcopenia in patients with CP. This review aims to investigate the prevalence and impact of sarcopenia in CP.

**Methods:**

Embase and Medline databases were used to identify all studies that evaluated sarcopenia and outcomes in patients with chronic pancreatitis. Due to paucity of data, conference abstracts were included. PRISMA guidelines for systematic reviews were followed.

**Results:**

Six studies, with a total of 450 individuals were reviewed. Three full-text studies and three conference abstracts met the predetermined eligibility criteria. The prevalence of sarcopenia in CP from all studies ranged from 17–62%. Pancreatic exocrine insufficiency was associated as an independent and significant risk factor for sarcopenia. Sarcopenia was found to be associated with a reduced quality of life, increased hospitalisation, and reduced survival. It was associated with significantly lower islet yield following total pancreatectomy with islet auto transplantation in CP.

**Conclusion:**

The review of these existing studies amalgamates the limited data on sarcopenia and its impact on CP. It has shown that sarcopenia is exceedingly prevalent and an important risk factor in CP patients. The data presented emphasises that sarcopenia has a significant prognostic value and should be included in future prospective analyses in the outcomes of CP.

## Introduction

Malnutrition is a common sequela of chronic pancreatitis (CP). Variations in body composition and the assessment of sarcopenia have gained the interest of clinicians in recent years. Different body composition counterparts are being assessed in various patient populations and sarcopenia is valued as a prognostic factor of morbidity and mortality. Sarcopenia is defined by low levels of measures of muscle strength, muscle quantity/quality and physical performance as an indicator of severity [1]. Sarcopenia is correlated with an increased risk of negative consequences such as physical disability, poor quality of life and death [2–4]. There has been more awareness and interest in sarcopenia of late due to its adverse influence on outcomes [5–7]. 'Primary' sarcopenia is defined as the loss in muscle mass and muscle strength when no other cause is evident but ageing itself [2, 8]. 'Secondary' sarcopenia is defined as the loss of muscle mass and muscle strength that accompanies underlying chronic diseases, advanced malignancies and malnutrition [2, 9, 10]. Sarcopenia and serum albumin levels are reported to be intimately linked with pancreatic exocrine insufficiency (PEI) in patients with CP [11].

Chronic pancreatitis occurs when repeated episodes of inflammation results in the replacement of pancreatic parenchyma with fibrotic connective tissue [12–14]. The recognised hallmark of advanced CP includes atrophy and fibrosis of the pancreas, distortion of ductal anatomy, strictures, and calcifications, which can result in impairment of both endocrine and exocrine functions [15, 16].

The quantification of muscle attenuation in clinical practice is a developing field of interest. Current available data on the impact of sarcopenia on the prevalence and outcomes in CP are scarce with only a handful of studies reporting on sarcopenia in pancreatic ductal adenocarcinoma (PDAC). We performed a review of the literature to investigate the prevalence and the impact of sarcopenia in patients with CP.

## Methods

Medline and EMBASE databases search was performed to identify studies, which evaluated the association of sarcopenia and CP. Medical Subjects Headings (MeSH) terms used include: ‘sarcopenia’, ‘chronic pancreatitis’, ‘exocrine pancreatic insufficiency’, ‘prevalence’, ‘outcomes’, and ‘mortality’. The search duration performed was from January 2010 to December 2019. The search was restricted to English-language studies. PRISMA guidelines for reviews were followed.

Eligibility criteria included adults over 18 years, studies detailing method of measurement of sarcopenia in CP, prevalence of sarcopenia detected in patients with CP, and outcomes of sarcopenia in patients with CP. The outcomes included any impact on CP reported, i.e. any morbidity or mortality related. The exclusion criteria were studies that did not report on the method of measurement of sarcopenia.

All relevant studies were screened by the title and abstract. All available full text studies were assessed in detailed. The reference lists were reviewed to identify additional appropriate articles. Due to the limited number of available studies on this topic, conference abstracts were included in the review. Two researchers carried out data collection, assessed the risk of bias and analysis independently. Any disagreements were resolved through discussion between the two reviewers or further adjudication by a third reviewer. The Newcastle–Ottawa Quality Assessment Scale was used for the assessment of the quality of the studies. The primary aims of the review were to identify the prevalence and impact of sarcopenia in patients with CP.

### Data collection

The data design of each study was retrieved with a predefined protocol for data extraction. The data captured included relevant information on key study characteristics, patient demographic profile, method of measuring sarcopenia, prevalence, and clinical outcomes (any). The intention was to analyse any similarities in negative outcomes between the studies, however, the heterogeneity of the findings precluded this.

## Results

**Fig. 1:**
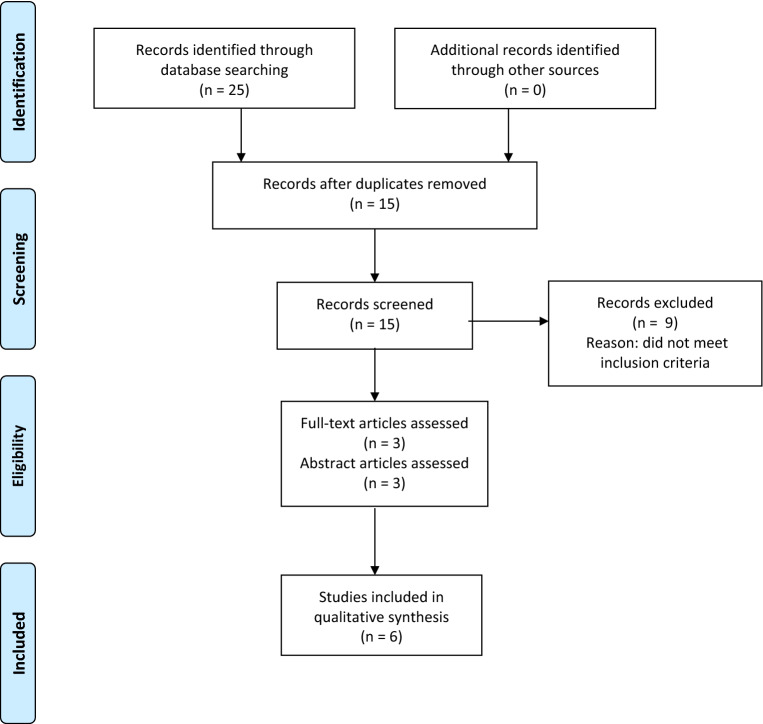
PRISMA flow diagram on Sarcopenia and Chronic Pancreatitis

The search produced 25 studies, and they were identified by title and abstract. After duplicates were removed, 15 studies were screened. Nine studies did not meet the eligibility criteria and were excluded. Three full-text studies and three conference abstracts fulfilled the eligibility criteria. The PRISMA diagram (Fig. 1) outlines the selection process. Six studies, which comprised of a total population of 450 patients, were reviewed (Table 1). There were two retrospective studies [17, 18] and four [11, 19–21] prospective studies. All studies were performed in a single institution. The studies reported heterogeneous outcomes and these are reported separately below. The outcome variations may well be the reality of clinical practice, but they made comparisons difficult.

**Table 1:** Characteristics of studies examining the prevalence and impact of sarcopenia in chronic pancreatitis

Study	Title	Year	Study type	Location	CP population	Mean age	Newcastle–Ottawa Score#
Olesen et al. [19]	Sarcopenia associates with increased hospitalisation rates and reduced survival in patients with chronic pancreatitis	2019	Prospective, single institution	Aalborg, Denmark	182	57.4 ± 12.9 years	9
Shintakuya et al. [11]	Sarcopenia is closely associated with pancreatic exocrine insufficiency in patients with pancreatic disease	2016	Prospective, single institution	Hiroshima, Japan	132 (9 with CP) = 6.8%	73 years (median)	7
Bieliuniene et al. [20]*	CT- and MRI-Based Assessment of Body Composition and Pancreatic Fibrosis Reveals High Incidence of Clinically Significant Metabolic Changes That Affect the *Quality of Life and Treatment Outcomes* of Patients with Chronic Pancreatitis and Pancreatic Cancer	2019	Prospective, single institution	Lithuania	63 (CP) 37 (PDAC)	58 years	6
Trikudanathan, et al. [21] (conference abstract)	Pre-Operative Sarcopenia Predicts Low Islet Cell Yield Following Total Pancreatectomy with Islet Autotransplantation (TPIAT) for Chronic Pancreatitis	2019	Prospective, single institution	Minneapolis, United States	138	38 years	N/A
O'Connor et al. [18] (conference abstract)	Investigating the prevalence of sarcopenia in chronic pancreatitis in an Irish cohort: A CT-scan based pilot study	2014	Retrospective, single institution	Dublin, Ireland	29	43 years (median)	N/A
Bulanova et al. [17] (conference abstract)	CT assessment of sarcopenia in patients with pancreatic cancer and chronic pancreatitis	2012	Retrospective, single institution	Moscow	29 (CP) 20 (PDAC)	29–63 years	N/A

^*^The study population size was 100, which included both CP and PDAC patients

*PDAC *Pancreatic ductal adenocarcinoma

^#^NEWCASTLE—OTTAWA QUALITY ASSESSMENT SCALE

### Definition of CP and measurement of sarcopenia

A diagnosis of CP was included as defined by the M-ANNHEIM classification system [22] in the study by Olesen et al. [19]. The other studies did not report how CP was assessed. Measurement of sarcopenia was performed in five studies at the level of the third lumbar vertebra (*L*3) using computed tomography (CT). The total cross-sectional areas of numerous muscles were quantified, which included the transversus abdominis, external and internal oblique abdominal muscles, rectus abdominis, psoas, erector spinae, and quadratus lumborum. A validated software tool was used for body composition analysis. The study by Olesen et al. measured sarcopenia with anthropometrics and bioelectric impedance (muscle mass), hand grip (muscle strength), and 'up and go' test (muscle function) [19]. The results on the measurement, prevalence, body mass index (BMI), and muscle mass index of sarcopenia in patients with chronic pancreatitis are shown in Table 2.

**Table 2:** Results of studies on the measurement, prevalence, BMI, and muscle mass index of sarcopenia in patients with chronic pancreatitis

Study	Measurement of sarcopenia	Number or patients with sarcopenia (prevalence) *N* (%)	Mean BMI (SD), kg/m2	Sarcopenia (BMI < 18)	Sarcopenia (BMI > 25)	Median L3 muscle mass index (cm2/m2)
Olesen et al. [19]	Anthropometrics Bioelectric impedance, Hand grip, Up and go test	31 (17.0%)	20.9 ± 4.1	8	–	–
Shintakuya et al. [11]	CT axial images at L3 vertebral	37 (15%)	–	–	–	43.51 (male) 36.26 (female)
Bieliuniene et al. [20]	CT axial images at L3 vertebral	21 out of 63 patients (33.3%)	24.08 ± 4.5	6 / 21 (29%)	3 /21 (14%)	49.60 ± 7.5 (men)47.00 ± 8.6 (women)
Trikudanathan et al. [21]	CT axial images at L3 vertebral	46 out of 138 patients (33.3%)	–	–	–	–
O'Connor et al. [18]	CT axial images at L3 vertebral	15 out of 29 patients (52%)	25.6	6	7	–
Bulanova et al. [17]	CT axial images at L3 vertebral	18 (62%)	22.16 ± 2.3	3/18	2/18	–

### Pancreatic exocrine insufficiency (PEI)

The faecal elastase or faecal fat test was used as a measure of the exocrine pancreatic function [19, 20]. PEI was defined as a fat excretion (aliphatic carboxylate [C14-C26]) > 25 mmol per 24 h or faecal elastase-1 level < 200 mg/g. Another alternative, ^13^C-labeled mixed triglyceride breath test was used and a percentage of ^13^CO_2_ cumulative dose at 7 h below 5% confirms PEI [11]. Two studies demonstrated PEI as a significant and independent risk factor for sarcopenia [11, 19]. Olesen et al. demonstrated that sarcopenia has a statistically significant association with opioid treatment (*p* = 0.045) and PEI (*p* = 0.03) on multivariate analysis [19]. An association between sarcopenia and PEI (*p* < 0.001)was displayed by Shintakuya et al. [11]. It should be noted that the results from this study, included all pancreatic diseases (malignancy, neuroendocrine, and CP) [11].

### Prevalence

The prevalence of sarcopenia from all studies ranged from 17–62% [11, 17–21].

### Outcomes

The outcomes of sarcopenia in patients with CP are shown in Table 3. The quality of life (QOL) was assessed using the European Organization for Research and Treatment of Cancer Quality of Life Questionnaire-C30 (EORTC QLQ-C30) [23]. Bieliuniene et al. demonstrated that CP patients had notably more extensive pancreatic fibrosis (PF) (*p* < 0.001) and sarcopenia decreased QOL in CP patients [20]. Olesen et al. also showed that sarcopenia was associated with reduced QOL, increased hospitalisation (*p* = 0.07), and reduced survival (*p* = 0.005) [19]. Sarcopenia was associated with significantly lower islet yield and more peri-operative blood loss (*p* = 0.002) following total pancreatectomy with islet autotransplantation in CP (*p* = 0.001) [21].

**Table 3:** Results on the impact of sarcopenia in chronic pancreatitis

Study	Impact of Sarcopenia in CP	Hospitalisation	Mortality	Quality of life (QoL)
Olesen et al. [19]	PEI was an independent risk factor for sarcopenia (*p* = 0.03). Sarcopenia was significantly associated with opioid treatment (*p* = 0.045) and PEI (*p* = 0.03) on multivariate analysis	Increased risk of hospitalisation (*p* = 0.07), increased number of in-hospital days (*p* < 0.001). Sarcopenia was not associated with an increased *risk of pancreatitis related* hospitalisation during the follow-up period (*p* = 0.39)	Reduced survival (*p* = 0.005)	Decreased median (IQR) global health scores (*p* = 0.003)
Shintakuya et al. [11]	Sarcopenia was associated with PEI in men (*P* < 0.001) and women (*P* = 0.012) on univariate analyses. Only sarcopenia remained independently associated with PEI (*P* < 0.001) on multivariate analysis.**takes into accounts all pancreatic diseases (malignancy, neuroendocrine, and CP)	N/A	N/A	N/A
Bieliuniene et al. [20]	There was no significant difference in sarcopenia status among patients with CP and PDAC (*p* = 0.85). The presence of osteopenia/osteoporosis predicts the presence of sarcopenia (*p* = 0.02). Patients with CP had more pronounced pancreatic fibrosis (PF) (*p* < 0.001).** takes into accounts pancreatic malignancy	N/A	N/A	Lower QOL in patients with PF ≥ 50% and in the CP group. (*p* < 0.001)
Trikudanathan et al. [21]	Peri-operative blood loss was more common in sarcopenic patients as compared to non-sarcopenic patients (*p* = 0.002). Sarcopenia was associated with significantly lower islet yield following TPIAT (*p* = 0.001).	N/A	N/A	N/A
O'Connor et al. [18]	This preliminary study has shown a high prevalence of sarcopenia in CP, independent of BMI	N/A	N/A	N/A
Bulanova et al. [17]	Sarcopenia is highly prevalent in patients with pancreatic cancer and CP (66%) and may be present in patients with any BMI values	N/A	N/A	N/A

## Discussion

This is a first review to examine the prevalence and impact of sarcopenia in patients with CP. In this review, sarcopenia has a prevalence of 17–64% in patients with CP. It has shown to have a statistical negative impact on various outcomes such as a lower QOL in CP patients with more than 50% pancreatic fibrosis, hospitalisation burden, mortality, and a lower islet cell yield following TPIAT.

Our findings indicate a paucity of research focusing specifically on sarcopenia and CP. We also found that there was major heterogeneity in the outcomes reported; however, the authors thought that all of the outcomes were important and had achieved statistical significance within respective studies. The included studies clearly support the hypothesis that sarcopenia negatively influence the outcomes of patients with CP. There is however limited evidence to provide a meaningful analysis in this review.

Sarcopenia is diagnosed when there is confirmation of low muscle quantity or quality; and is considered severe in addition of low muscle strength, and poor physical fitness [1]. The process of muscle tissue loss commences approximately at 40 years of age and progresses at a rate of 8% loss of muscle tissue per decade until the age of 70, which then accelerates to 15% thereafter per decade [24]. The pathophysiology of sarcopenia is complex and is attributable to reduction in caloric consumption, denervation of muscle fibres, intracellular oxidative stress, hormonal decrease, and enhanced myostatin signalling [9]. This review has demonstrated that sarcopenia is prevalent in patients with CP and may be present in underweight, normal weight and obese patients. It is important to diagnose sarcopenia regardless of the BMI, as the presence of sarcopenia did not correlate with BMI values. Individuals of similar BMI display different percentages of lean and adipose tissue [25, 26]. Sarcopenic obesity is defined as a reduction in lean body mass in the context of excess fat [27], and it is easily overlooked in obese patients. Individuals who are obese and sarcopenic have worse outcomes than those who are sarcopenic and non-obese [28]. Olesen et al. reported that 23(74%) of sarcopenic patients had a normal or obese BMI and demonstrated a significant association between sarcopenia and BMI subgroups (*p* = 0.009) [19].

The specificity and precision of body composition measurement modalities offer a new perspective to view the body habitus. Each has its own pros and cons in assessing changes in muscle or adipose tissue distribution. They provide a robust assessment for sarcopenia which is simple, feasible, and help facilitate development of comprehensive approaches to decision-making regarding peri-operative care [29]. Conventional anthropometric parameters have a low index in detecting sarcopenia; however, it is not an accurate assessment of muscle tissue [30].

Conventional nutritional assessments also do not accurately detect sarcopenia, and radiology has been proven to be more reliable [31]. The utilisation of radiology in the examination of body composition is highly differentiated, with the technology to recognise and discriminate between muscle, fat, as well as the distribution of adiposity within intermuscular, subcutaneous, and visceral sites [25]. Dual-energy X-ray absorptiometry (DXA) is feasible, accurate, non-invasive, inexpensive, and regarded as the ideal standard for quantifying muscle mass [32]. However, the gold standards for non-invasive measurement of muscle mass are magnetic resonance imaging (MRI) and CT [33]. The evaluation of sarcopenic obesity using CT data was introduced in 2013 [34], and is an objective and precise assessment of sarcopenia. The cut-off points for the diagnosis of sarcopenia are arbitrary at this time; however, Prado et al. defined sarcopenia cut-offs at approximately 52.4 cm^2^/m^2^ for men and 38.5 cm^2^/m^2^ for women [25]. The use of imaging to evaluate sarcopenia however does not assess the aspect of functional muscle status.

An alternative of measurement of body muscle/fat composition is a hand-held bioelectric impedance analysis (BIA) machine that is affordable, widely available, and portable. It procures an evaluation of muscle mass based on whole-body electrical conductivity [1]. In non-obese adults, an accurate two-compartment (lean, fat) measurement of body composition can be made in 10 min with a BIA machine [35]. The studies in this review used CT imaging to detect sarcopenia and only one study used anthropometric measures. It should be noted, however, that sarcopenia is a relatively subjective measurement.

It is worth mentioning the impact of sarcopenia in pancreatic ductal adenocarcinoma (PDAC). Bieliuniene et al. included patients with PDAC  and reported that a substantial number of cases of sarcopenia were detected in patients with CP (62%), demonstrating that sarcopenia was more prevalent in chronic diseases in contrast to malignancy [20]. Shintakuya et al. took into account all pancreatic diseases (malignancy, neuroendocrine, and CP) [11]. Although they were analyzed separately in subgroups, this is a potential confounding variable. Recent meta-analyses have shown sarcopenia (HR 1.49; 95%CI 1.27–1.74, *p* < 0.001) and sarcopenic obesity (HR 2.01; 95%CI 1.55–2.61, *p* < 0.001) are significantly associated with worse overall survival in patients with PDAC [36]. A study has shown that sarcopenia is a strong predictor of the occurrence of pancreatic fistula and survival after pancreatoduodenectomy and recommends reconditioning of the sarcopenia prior to the operation [37].

Patients with CP potentially have a more pronounced level of malnutrition. Various factors such as intractable pain, alcoholism, malabsorption, and maldigestion from PEI, renders these patients at a considerable risk for sarcopenia. Patients with CP are likely to have more co-morbidities which may contribute to the loss of muscle mass and more adverse outcomes. CP was not properly defined except for one study which used the M-Anheim assessment [19]. Nutritional assessment and the complications of CP are pivotal in the management of these patients. Approximately 30%–50% of CP patients have increment of resting energy expenditure [38]. The key strategy in these patients is the early detection of sarcopenia and active interventions. The management involves a multidisciplinary team and includes alcohol abstinence, pain management, nutritional and dietary improvement, and pancreatic enzyme supplementation [39].

At this point in time, there is no evidence to suggest that sarcopenia can be treated or is modifiable. There is insufficient evidence available to guide the treatment of sarcopenia. In a complex disease like CP, an optimal body habitus is unlikely to be achievable in the short term. Nevertheless, the prognostic value of sarcopenia can be a valuable adjunct. Persons with sarcopenic obesity were evaluated with various exercise interventions (aerobic, resistance, and combined), and it was discovered that those in the resistance group showed the most improvements in strength [40]. The very first randomised controlled trial (RCT) is currently under study by the Japanese which evaluates the clinical influence of exercise therapy on sarcopenia in CP patients [41]. We await the results with anticipation.

Despite the relevant findings, this review has a few limitations. First, due to the paucity of data, conference abstracts were included to provide a more robust data for the review. The studies analysed were retrospective and prospective cohorts, and consisted of heterogeneous patient populations hence selection bias may be a limitation. All studies were single-centred, and consisted of a small patient population hence within each study, analysis remains a potential limitation. The large range of prevalence of sarcopenia reported across the studies may be due to how it was measured (large vs small population) and the duration the patients had been diagnosed with CP before they entered into the study.

Nevertheless, this review provides a platform to expand on this important prognostic factor. An increased effort is required to address the deficiency of research on this topic as evident in Table 3. Future studies should be designed to analyse the relevance of sarcopenia on the prognosis and management of CP patients by investigating the negative clinical outcomes. In addition, there is also a need for serial assessments of patients prospectively while attempting to treat their sarcopenia.

## Conclusion

The review of these existing studies amalgamates the paucity of data on sarcopenia and its impact on CP. It has shown that sarcopenia is exceedingly prevalent and an important risk factor in CP patients. The data presented emphasises that sarcopenia has a significant prognostic value and should be included in future prospective analyses on the outcomes of CP. There is a crucial need for more studies to address the deficiency of research on this topic and further elucidate the impact of sarcopenia in patients with CP.
